# Assessment of Heavy Metal Accumulation in Soil and Garlic Influenced by Waste-Derived Organic Amendments

**DOI:** 10.3390/biology11060850

**Published:** 2022-06-01

**Authors:** Pervaiz Akhter, Zafar Iqbal Khan, Muhammad Iftikhar Hussain, Kafeel Ahmad, Muhammad Umer Farooq Awan, Asma Ashfaq, Usman Khalid Chaudhry, Muhammad Fahad Ullah, Zainul Abideen, Khalid S. Almaary, Mona S. Alwahibi, Mohamed Soliman Elshikh

**Affiliations:** 1 Department of Botany, University of Sargodha, Sargodha 40100, Pakistan; pervaizakhter70@gmail.com (P.A.); zafar.khan@uos.edu.pk (Z.I.K.); kafeeluaf@yahoo.com (K.A.); asma.ashfaq@uos.edu.pk (A.A.); 2 Department of Plant Biology & Soil Science, Universidad de Vigo, 36310 Vigo, Spain; 3 Department of Botany, Government College University, Lahore 54000, Pakistan; mufawan@gmail.com; 4 Institute of Soil and Environmental Sciences, University of Agriculture Faisalabad, Punjab 38000, Pakistan; ukojla0455@gmail.com; 5 Department of Earth Sciences, University of Sargodha, Sargodha 40100, Pakistan; muhammad.fahadullah@uos.edu.pk; 6 Dr. Muhammad Ajmal Khan Institute of Sustainable Halophyte Utilization, University of Karachi, Karachi 75270, Pakistan; zuabideen@uok.edu.pk; 7 Department of Botany and Microbiology, College of Science, King Saud University, Riyadh 11451, Saudi Arabia; almaary786@gmail.com (K.S.A.); malwhibi@ksu.edu.sa (M.S.A.); melshikh@ksu.edu.sa (M.S.E.)

**Keywords:** heavy metal, organic manures, garlic, health risk

## Abstract

**Simple Summary:**

The utilization of organic wastes, for example, poultry waste, sugarcane press mud, and farmyard manure is extremely common among farmers from South Asia including Pakistan. We assess the biochemical nature and dietary benefit for plants and record the presence of heavy metals in garlic following cultivation in a soil amended with these organic wastes. Present investigation showed the grouping of toxicity and bioaccumulation of heavy metals with a particular link to their source of origin and highlight the hazard of some of them for public health through their excessive utilization. Selecting appropriate manure can significantly reduce health risks for humans.

**Abstract:**

In South Asia, the high costs of synthetic fertilizers have imposed research on alternative nutrient inputs. We aimed to identify potential trace elements (PTE) present in some organic manure that might be a source of environmental pollutions and risk to public health following consumption. The study aims to evaluate how different organic waste (poultry waste, PW; press mud, PM; and farmyard manure, FYM) influences the heavy metal migration in soil, the accumulation in garlic, and their potential health risks. Organic waste caused a higher accumulation of certain metals (Zn, Cu, Fe, and Co), whereas Mn, Cd, Cr, and Pb were in lower concentrations in soil. Amendments of soil with PM revealed a higher accumulation of Cd, Cr, Fe, and Pb, whereas PW resulted in Cu and Zn accumulation in garlic. Treatment of soil with FYM exhibited higher metal concentration of Co and Mn. An environmental hazard indices study revealed that pollution load index (PLI) was highest for Cu following treatment with PM. The health risk index (HRI) was greater for Cd following amendment with PM. Maximum daily intake of metals (DIM) was observed for Zn after treatment with PW. The Pb exhibited maximum bioconcentration factor (BCF) in PM-amended plants. Based on these findings, we concluded that garlic grown on contaminated soil with organic waste may pose serious health hazards following consumption.

## 1. Introduction

Globally, soil heavy metal contamination is a major issue influencing vegetable production, especially near urban areas. Natural and anthropogenic activities continually degrade the soil and increase soil heavy metal pollution. High concentrations of heavy metals strongly affects the soil, leading to heavy metal toxicity [1]. Plants, being sessile, need to survive under natural conditions and the uptake of water and nutrients for their normal growth functions are important; however, heavy metal toxicity forces their uptake by vegetables and deposits in edible parts [2]. The accumulation of heavy metals in plant tissues and fruit disrupts the nutritional quality and it becomes the part of food chain. The consumption of such vegetables poses serious threats to humans as well as animals [3].

Concentration of trace metals can be determined by comparing consumption of vegetables on a daily basis for humans. It is the ideal approach to evaluate the relative contrasts between a concentration of toxic metals in farmland and in vegetables [4]. A higher incentive for an exchange remainder shows that vegetables have higher potential for the accrual of toxic metals [5]. Upper layers of soil contain excessive heavy metals that are easily taken up by plants in contrast to essential nutrients. Vegetables grown on contaminated soils may cause cardiovascular infections, kidney problems, sensory system issues, and bone and joint maladies [6].

Organic amendments supply macro- and micro-nutrients, as well as carbon, to help restore the physical and chemical qualities of soil. The presence of heavy metals and the inability to control the transformations required to convert organic forms of N and P into minerals available to crops, particularly to minimize losses of these nutrients in forms that may pose a threat to human health, and thus exhibit several challenges due to the use of organic amendments [7,8,9,10], is challenging. Animal manure and sewage biosolids, which are the most abundant organic amendments, contain components that are potentially harmful to human health, animals, and plants [7,8,9,10]. Currently, applications of different waste-derived organic amendments in vegetable–soil systems have the potential to improve the quality and profitability of vegetables grown on contaminated soils. Additionally, it minimizes negative influence of inorganic fertilizers to ensure supply of healthy vegetables [7,8,9,10]. Organic manures that can be used as substitutions for inorganic fertilizers include farmyard manure, poultry waste, and press mud, all of which contain a lower quantities of heavy metals. Press mud is a by-product of sugarcane industries, and is easily available in Pakistan, due to its higher production of 6.1 million tons. It can be employed as a potential source for the remediation of heavy metal contamination [11]. Additionally, in Pakistan, poultry production has expanded to also produce by-products such as poultry waste. The application of organic manure reduces the mobility with the least deposition of traces metals in soil and its uptake by plants [12]. Animal waste harbors essential natural supplements for vegetables’ growth and production. It can be extensively used as an organic amendment in non-formal cultivation for improving the production of vegetables [13]. 

Garlic (*Allium sativum* L.) originated in Central Asia, but currently is grown worldwide. It is a vegetable crop, the bulb and shoot being the edible parts [14]. It is used as condiment vegetable and in sauces to enrich the flavor of food. It is rich source of volatile and non-volatile compounds, nitrogen oxides, proteins, flavonoids, saponins, sapogenins, phenolic compounds, antioxidants, minerals (Se, P and K), and vitamins B and C complexes [15]. Meanwhile, dry bulbs of garlic have 62–68% water, 26–30% carbohydrates, and 1.5–2.1% protein [16]. In ancient times, it was also used a medicinal crop for the treatment of headaches, heart problems, worms, and tumors. It is at a risk of heavy metal contamination due to several anthropogenic activities [17].

The Sargodha is a major agriculture- (wheat, rice, sugarcane, fodders, vegetables, and citrus) producing region in Punjab, Pakistan. Different organic amendments such as poultry waste, sugarcane press mud, and farmyard manures are extensively used in agriculture to increase crop yield [3,5,8,9,11]. However, the fate of heavy metals (e.g., Cr, Pb, Co, As, Cu, and Cd) deposited from such practices has not been sufficiently investigated. Different vegetables’ responses to heavy metal contamination was previously determined with the application of sewage water [18]. Another study reported the growth performance of garlic with the alleviation of Cd by silicon application [19]. However, according to our knowledge, there is a lack of information related to garlic growth in response to deposition of heavy metals in farmland soils following organic amendments. Therefore, the present study was conducted to evaluate if any of the possible trace elements (PTE) found in some organic manure could be a cause of pollution or pose a concern to public health following vegetable (garlic) consumption. The specific aims were to (a) evaluate the effects of application of poultry waste, press mud, and farmyard manure on garlic growth, pollution load, and heavy metals accumulation in soil and vegetable; (b) assess the human health risk induced from soil exposure and food consumption; (c) determine the risk induced by different organic manure-borne heavy metals; and (d) estimate the soil/vegetable contamination risk to public health posed by organic fertilizer applications. This study will bridge the gap of limited information available from waste-derived organic amendments, and understand the estimation of health risk and whether their application influences the toxic metals accumulation in soil and garlic and.

## 2. Materials and Methods

### 2.1. Study Area

The current study was conducted during the year 2017 (January to April) in the northeast area of Pakistan. The precipitation rate in the region is approximately 175–210 mm with an average temperature of 6–24 °C during the winter season and 25–49 °C during the hot summer season.

### 2.2. Experiment

A pot study was executed for the evaluation of agronomic characteristics of garlic by amending heavy metal contaminated soil with the application of organic manures such as farmyard manure, poultry waste, and sugarcane press. Ten seeds of garlic were sowed in each pot having clay and loamy soil amended with the treatment of control (C), poultry waste (PW), farmyard manures (FYM), and press mud (PM). Each pot was irrigated with tap water until the first compound leaf appeared. Environmental conditions during the experiment consisted of an average temperature of 25 °C during the day and 20 °C during the night, with a relative humidity of 55–60%. Collection of organic manure was done from sub-Saharan areas of Sargodha, Pakistan. Organic manures (PW, FYM, and PM) were added to each pot in three replicates and a total of 12 pots were designated for each treatment by following a completely randomized design (CRD). All the organic manures were applied to each pot as 1:1 (50% organic manure + 50% loamy clay garden soil). Garlic plants were harvested at two intervals. The first harvest was taken 45 days after the emergence of seedlings and the second harvest was approximately 90 days after for the collection of bulbs.

### 2.3. Sample Collection

#### 2.3.1. Soil and Plant Sampling

Soil sampling was done randomly from all the treated pots. The sample were dried, crushed, and sieved with a 2 mm screen before being sealed in plastic bags and kept at −80 °C until analysis. Garlic samples were collected and immediately washed with distilled water and later with diluted hydrochloric acid for removal of dust particles and other impurities. Plant samples were washed with deionizer water to remove the visible soil and samples were separately crushed in grinder and stored at −80 °C for further analyses. For analysis, samples were air dried for 4 days and stored in sealed paper bags and complete drying was done in an oven at 80 °C for 3 days.

#### 2.3.2. Wet Digestion and Spectroscopic Analyses

The wet digestion method was employed for the digestion of soil and plant samples. The incubated soil and plant samples were digested by following the wet digestion method. For the estimation of heavy metals concentration, approximately 1.0 g of completely air-dried soil and plants were deposited into a digestion flask. After that, 5 mL of concentrated HNO_3_, HClO_4_, and H_2_SO_4_ (5:1:1) was added to the samples and kept for 24 h for complete digestion. The mixture was left to cool and filtration was done by Whatman filter paper #42. Metals concentration in experimental soil and garlic leaf samples were analyzed by an atomic absorption spectrophotometer. Metal ions estimated in this study were Cd, Cr, Cu, Co, Fe, Mn, Pb, and Zn. Analytical methods for AAS were conducted by following standard procedure as explained by the protocol guide of the European Commission [20]. Limit of detection (LOD) rates were also estimated by following standard methods explained by [21]. 

### 2.4. Quality Control

Calibration of the apparatus was done by using standard values to ensure quality control. In this way, deionized water was used throughout the work and crystal pupillages were cleaned. Authentication of the constant results was done by value declaration and by evaluating consistent Specialized Position Quantifiable (SRM-2711 for soil and SRM NIST 1577b for garlic). The average recoveries of the SRM for soil were Pb 104%, Cu 96%, Co 93%, Mn 92%, Cd 95%, Cr 98%, and Zn 90%, respectively, and the average recoveries of SRM for the garlic were Pb 95%, Cu 91%, Co 94%, Mn 109%, Cd 92%, Cr 97%, and Zn 88%.

#### 2.4.1. Pollution Load Index (PLI)

It was measured by following the equation described below [22].

$$\mathrm{PLI}=\frac{\mathrm{metal}\;\mathrm{in}\;\mathrm{soil}\;\mathrm{sample}}{\mathrm{metal}\;\mathrm{in}\;\mathrm{reference}\;\mathrm{sample}}$$

where the background sample values were Cd (1.49 mg kg^−1^), Cr (9.07 mg kg^−1^), Cu (8.39 mg kg^−1^), Fe (56.90 mg kg^−1^), Ni (9.06 mg kg^−1^), Mn (46.75 mg kg^−1^), and Zn (44.19 mg kg^−1^).

#### 2.4.2. Bioconcentration Factor (BCF)

It is known as the accumulation of metals by plants with the uptake of heavy metals from contaminated soils. It was estimated by the given formula [23].

$$\mathrm{BCF}=\frac{C_{\mathrm{veg}}}{C_{\mathrm{soil}}}$$

where C_veg_ is accumulation of metal in plant tissues (mg kg^−1^) and C_soil_ is deposition of metals in soil (mg kg^−1^).

#### 2.4.3. Daily Intake of Metals (DIM)

It was measured by following the method provided by [24]. It is used to assess risk to consumer health.

$$\mathrm{DIM}=\frac{C_{\mathrm{metal}}\times D_{\mathrm{food}\;\mathrm{intake}}}{B_{\;\mathrm{average}\;\mathrm{weight}\;}}$$

where C_metal_ shows concentration of metals in grains, D_food intake_ shows daily intake of food, and B_average weight_ shows average body weight. The average daily vegetable intake rate of adult is 0.345 kg person^−1^ day^−1^ reported by [23].

#### 2.4.4. Health Risk Index (HRI)

It refers to the daily intake of metal in any food [25]. It was measured by following the standard equation given below.

$$\mathrm{HRI}=\frac{\mathrm{DIM}}{\mathrm{RfD}}$$

where DIM is daily intake of metals and RfD is the oral reference dose. HRI > 1 of any metal in food crops indicates high health risk.

### 2.5. Statistical Analysis

Data were analyzed using one-way analysis of variance (ANOVA) using SPSS (20.0). The mean values and standard errors were calculated by using MS Excel (v2007). Principal component analysis (PCA) and cluster analysis were performed by using Origin 2021 (OriginPro, Version 2021, Origin Lab Corporation, Northampton, MA, USA).

## 3. Results and Discussion

### 3.1. Concentration of Metal Ions in Soil

Metal ions in soil samples ranged from 0.332 to 0.448 mg kg^−1^, 0.455 to 0.537 mg kg^−1^, 0.135 to 0.779 mg kg^−1^, 1.222 to 1.310 mg kg^−1^, 0.108 to 0.179 mg kg^−1^, 0.719 to 5.682 mg kg^−1^, 0.023 to 0.537 mg kg^−1^, and 4.535 to 17.633 mg kg^−1^ for Cd, Co, Cr, Cu, Fe, Mn, Pb, and Zn, respectively (Table 1). As a result of the poultry waste, press mud, and farmyard manure treatments, metals such as Zn, Cu, Fe, and Co exhibited higher concentrations whereas the metals Mn, Cd, Cr, and Pb were lower in concentration (Table 1). The application of organic manures resulted differentially for the accumulation of heavy metals. For instance, the accumulation of Cd, Co, and Cr metals was higher in the pressmud-treated pot. The Cu, Mn, and Zn metals showed higher deposition in soil in the poultry waste-treated pots, whereas the farmyard manure solely exhibited higher Fe accumulation. Analysis of variance results exhibited that the application of treatments had a non-significant (*p* < 0.05) effect on trace metal concentrations of Co, Cr, Pb, and Zn, whereas Cu concentration showed significant (*p* > 0.05) results (Table 2). Supplementation of organic fertilizers improves the soil characteristics as well as improves the organic matter contents of the soil [26]. Additionally, organic amendments contain organic acid anions that utilize proton during decomposition resulting in increased soil pH and nutrient availability [27]. The higher soil pH immobilizes the heavy metals via precipitation, ion exchange, and adsorption [28]. Our results are in accordance with [29], that organic fertilizer alleviated the harmful effects of heavy metals with reduced deposition in soil. Lower soil pH reduces the adsorption of oxides and minerals; therefore, it increases the availability of metal ions in soil. Contrarily, higher pH limits the availability of metal ions [30]. In this study, organic manures increased the pH of the soil. Therefore, reduction in mobility and bioavailability of the metal ions was noticed. Furthermore, [31] reported that soil pH disturbed the chemical forms of metal ions by disturbing their precipitation/dissolution, adsorption/desorption, and complex formation. Results obtained regarding decreased heavy metal concentration due to organic fertilizers application are also in accordance with [4]. Our results are also corroborated by an earlier study [32] that reported immobilization of heavy metals with the application of organic manure. The permissible maximum limits reported by [24] of the trace metals is Cd 3, Co 65, Cr 100, Cu 50, Pb 300, Fe 21000, Mn 2000, and Zn 200 mg kg^−1^ deposited in soil. All the treated pots showed lower metal concentrations than permissible limits.

**Figure 1:**
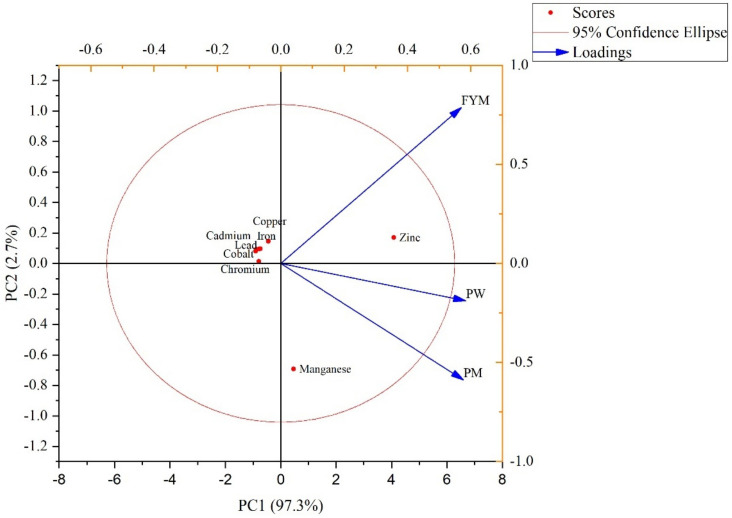
Principal component analysis of mean values of metal accumulated in soil samples. It is a is a combination of score plot of metal ions (represented as dots) and loading plot of treatments (represented as vectors). FYM: farmyard manure, PW: poultry waste, PM: press mud.

**Figure 2:**
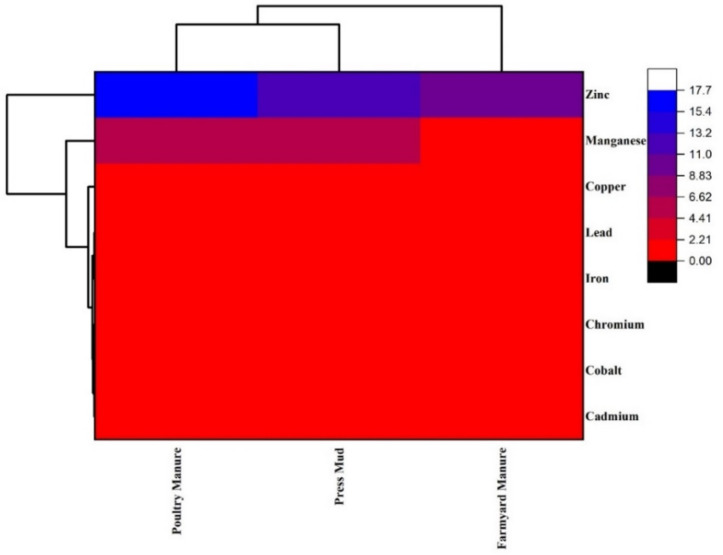
Dendrogram of 8 metals (copper, cobalt, chromium, cadmium, lead, iron, manganese, zinc) based on different treatment groups (poultry waste, press mud, and farmyard manure) from soil samples. It was generated with a hierarchical cluster analysis with Average Linkage (between groups).

The principal component analysis (PCA) was performed for all the metal ions mean data of all three treatments (PW, PM, and FYM) to observe component loading and the clustering behavior (Figure 1). The first principal component PC1 has 97.3% variance and the second component PC2 has 2.7% variance (Figure 2). The Cd, Cu, Co, Cr, Fe, and Pb lied in the negative quadrate, whereas Mn and Zn were in positive quadrate. The first group of variables with which PC1 is positively correlated includes: Zn and Mn. A significant negative correlation of PC1 variables was found with the variables aligned with PC2: Cd, Cu, Co, Cr, Fe, and Pb (Figure 1). The loadings of all metal ions except Mn and Zn suggested that they were strongly correlated to the same source of contamination in soil. The PCA results also showed that all the organic treatments contributed to the heavy metal pollution loads of the tested soil (Figure 1).

A hierarchical cluster analysis with Average Linkage (between groups) was used to observe similarity/variations in metal ions among the tested soil samples (Figure 2). The results shown in the dendrogram grouped all the metal ions in same clade except Mn and Zn. It grouped these two metals (Mn and Zn) separately, whereas the second main group, which included Cd, Co, Cr, Fe, Pb, and Cu, was grouped as a separate subgroup (Figure 2). It showed differential accumulation of Mn and Zn in soil compared with the other metal ions. It further strengthened the PCA results by grouping Mn and Zn separately, as their source of deposition in the soil was different from the remaining metal ions.

### 3.2. Heavy Metal Concentrations in Garlic Samples

#### Concentration of Metal Ions in Garlic

The current study also reported the accumulation of metal ions in the garlic. The range of concentration levels were from 0.444 to 0.495, 0.402 to 0.463, 0.169 to 0.381, 1.130 to 1.189, 0.187 to 0.966, 2.94 to 5.68, 0.052 to 0.501, and 4.53 to 18.28 mg kg^−1^ for Cd, Co, Cr, Cu, Fe, Mn, Pb, and Zn, respectively (Table 1). In different metals, the Zn concentration was the highest whereas Mn concentration was lowest in the pots treated with PW (Table 1). In garlic leaves, the higher accumulation of Cd, Cr, Fe, and Pb was attributed to PM- treated pots. The PW application showed higher concentrations of Cu and Zn, Likewise, FYM-treated pots exhibited a higher metal concentration of Co and Mn. The ANOVA results showed that the treatments had significant (*p* < 0.05) differences for Cu, Cd, Cr, and Pb, and non-significant (*p* > 0.05) results for the concentration of Co and Fe (Table 1 and Table 2). The permissible maximum limits reported by [33] of the metals were Cd 2, Co 50, Cr 50, Cu 20, Pb 10, Fe 425, Mn 500, and Zn 100 mg kg^−1^ accumulated in plants. Accumulation of heavy metal range in *A. sativum* was lower than the permissible maximum limits, except for Pb. Higher accumulation of Pb in *A. sativum* can be attributed to extensive application of pesticide or mineral fertilizers to the farmland of the collected soil for the study. Similar results were reported by [34] in garlic. The levels of Cu and Zn in *A. sativum* samples were higher than the values reported by [35]. Contrarily, the level of Cu was higher than the values reported by [36]. The mean values of Co, Cr, Mn, and Fe were found lower while Zn value was higher in contrast to the values previously reported [37]. Our results regarding higher Zn and Cu metal ions are also in agreement with the findings of [18]. It might be due to higher absorption of these metal ions in garlic plants.

**Figure 3:**
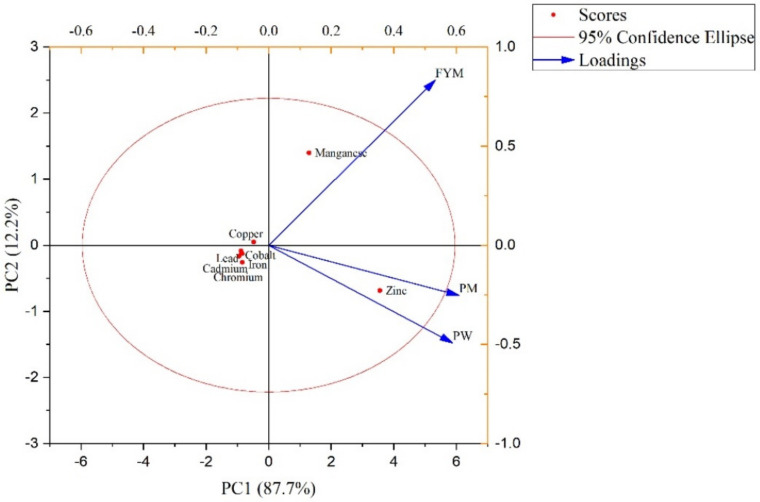
Principal component analysis of mean values of metal accumulated in garlic plants. It is a combination of score plot of metal ions (represented as dots) and loading plot of treatments (represented as vectors). FYM: farmyard manure, PW: poultry waste, PM: Press mud.

**Figure 4:**
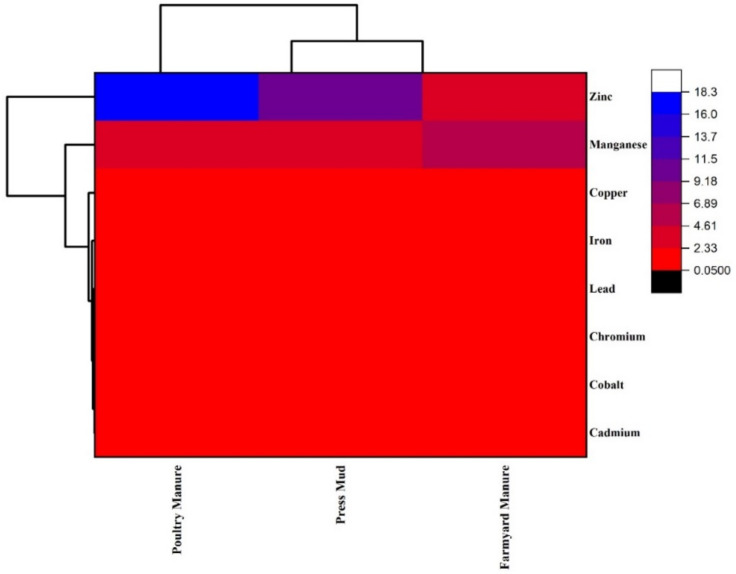
Dendrogram of 8 metals (copper, cobalt, chromium, cadmium, lead, iron, manganese, zinc) based on different treatment groups (poultry waste, press mud, and farmyard manure) from garlic plant. It was generated with a hierarchical cluster analysis with Average Linkage (between groups).

The principal component analysis was conducted to observe the clustering behavior of the garlic plant. The first principal component PC1 showed 87.7% variance and the second component, PC2, showed 12.2% variance (Figure 3). The PC2 has negative loadings of Cd, Co, Cr, Fe, and Pb, whereas Cu is close to the origin and showed inappreciable accumulation in garlic. The Mn accumulation was higher in response to FYM application, whereas Zn showed a higher deposition in garlic following poultry manure and press mud. The clustering of all metal ions except (Mn and Zn) suggested that they were strongly correlated. The PCA results clustering also revealed that organic amendments assisted in the suppression of heavy metal accumulation in garlic (Figure 4). The average linkage cluster analysis was used for dendrogram grouping to describe similarity/variations among metal ions in plant samples (Figure 4).

### 3.3. Bioconcentration Factor

Bioconcentration factors for all metals found lower in control pots compared with the treated pots. Metal ions ranged from 0.0348–25.76 for BCF values (Table 3). The Pb exhibited the maximum BCF in PM-amended pots, whereas minimum BCF was observed for Mn from PW-treated pots. The BCF values order for metals concentration for PW, PM, and FYM treatments were Mn < Zn < Co < Cu < Fe < Cr < Pb < Cd, Cu < Co < Mn < Fe < Cr < Zn < Cd < Pb and Co < Cu < Zn < Cr < Cd < Fe < Mn < Pb (Table 3). The BCF demonstrates the bioavailability of substantial metals at a specific area for various assortments of vegetables [10,38]. The current study exhibited the differential BCF value for each metal ion. The highest BCF observed for Pb, whereas lowest was for Mn. The BCF values of higher than one suggests that it can be easily available for plant uptake [23]. The BCF value of Cd, Fe, and Pb was higher than compared to other metal ions, therefore these metal ions accumulated in garlic. These results are also in line with our earlier study on peppers [10]. 

### 3.4. Pollution Load Index

The PLI value of metals in soil grown with *A. sativum* in control treatment was lower with PW-, PM-, and FYM-treated pots. Different metal ions showed differential PLI values, and it was higher for Cu treated with PM and lowest for Pb under control conditions. Metal ions ranged between 0.00061–0.7002 for PLI values. The order of PLI of different metals for was: PW, PM, and FYM Pb < Cu < Mn < Cd < Fe < Zn < Cr < Co, Fe < Cr < Pb < Co < Cd < Cu < Zn < Mn, and Pb < Co < Cr < Mn < Cu < Cd < Fe < Zn, respectively (Table 4). The PLI highlights the existence of contamination in soil [38]. It is known as the indicator of contaminated soil, as its value of less than one suggests that soil is free from any contaminant, whereas greater than one shows contamination of soil [39]. In our study, the PLI value was less than one for all the observed metals and shows that garlic was free from any contamination and consumable. Wajid et al. [40] reported the higher PLI values for the Cu, Cd, Fe, Mn, and Pb with the application of mineral fertilizers. Related results regarding lower PLI value were also reported by [41] from the collected soil samples.

### 3.5. Metal Intake Associated Health Risks

The maximum value of daily intake of heavy metals was predicted to be 0.2945 mg for Zn in PW treatment, whereas the minimum concentration of DIM for metals was likely to be 0.0003 for Mn in PW treatment. The range of HRI of *A. sativum* in differently treated pots was between 0.0006 and 2.89. HRI values of all treatments were in the following order: Cr < Mn < Fe < Co < Cu < Zn < Pb < Cd. Cd showed the highest HRI value treated with PM, whereas the lowest was Cr under control conditions (Table 5). The assessment of health risks through the food chain majorly concerns many developing countries where wastewater is used for vegetable production [42]. Heavy metals exposure in humans occurs with the intake of contaminated air, water, and food [43]. The HRI analysis of metal ions from soil and crops indicated that the HRI values of Pb, Cr, Cd, Ni, and Mn ranged from 18.3 to 66.7, 31.6–61.6, 7.13–11.1, 30.0–64.3, and 11.5–90.0, respectively, and within the permissible limits by [33]. The HRI values of Pb and Cd are hazardous and can cause health issues even at a low concentration [44]. High BCF for Cu, Co, and Zn with different treatments was observed in this study, whereas DIM values were lower when compared with DIM qualities for Fe (45), Zn (40), Cu (10), Ni (1), and Pb (0.240) (mg kg^−1^ day^−1^), as recommended by [42]. HRI relies on ecological factors for various vegetables and usage of organic wastes, pesticides, soil conditions, and different synthetic substances. Other researchers reported lower values for Zn (2.41 × 10^−3^), Cu (5.32 × 10^−2^), and Pb (7.75 × 10^−5^) compared with the present investigation [45].

## 4. Conclusions

The current study highlighted that the negative impact of different organic wastes (poultry waste, press mud, and farmyard manure) had resulted in greater pollution loads to soil and garlic. Organic amendments resulted in a higher accumulation of certain metals (Zn, Cu, Fe, and Co), whereas Mn, Cd, Cr, and Pb were lower in concentrations in soil. The amendment of soil with the PM application caused a higher accumulation of Cd, Cr, Fe, and Pb, whereas Cu and Zn accumulated in garlic. FYM showed Co and Mn contents. Similarly, the level of metal ions in soil samples was below permissible limits. Additionally, the metal accumulation in garlic plants also showed lower values than permissible maximum limits. Contrarily, health risk index values of Cd and Pb showed higher values than one that suggested the probability of health issues. It can be suggested that garlic grown on contaminated soil may pose serious health problems after its consumption. The environmental impacts of organic fertilizers, changes in soil fertility level, as well as how to sustain and improve them, are prerequisites and must be assessed in future works.

## Figures and Tables

**Table 1:** Heavy metal concentrations in soil and *Allium Sativum* L. (mg kg^−1^).

Metal (mg kg^−1^)	Treatments				MPL (mg kg^−1^)
	C	PW	PM	FYM	
**Soil**					
Cd	0.448 ± 0.003	0.332 ± 0.068	0.457 ± 0.004	0.394 ± 0.011	0.5
Co	0.537 ± 0.0026	0.455 ± 0.093	0.562 ± 0.056	0.488 ± 0.005	1
Cr	0.135 ± 0.014	0.146 ± 0.004	0.779 ± 0.006	0.189 ± 0.004	50
Cu	1.222 ± 0.006	1.310 ± 0.041	1.251 ± 0.006	1.254 ± 0.005	20
Fe	0.179 ± 0.015	0.126 ± 0.020	0.108 ± 0.016	0.141 ± 0.016	10
Mn	1.868 ± 0.759	5.682 ± 0.150	5.682 ± 0.150	0.7196 ± 0.013	1000
Pb	0.537 ± 0.006	0.023 ± 0.004	0.082 ± 0.032	0.0529 ± 0.027	30
Zn	4.535 ± 0.57	17.633 ± 1.120	12.69 ± 0.022	10.713 ± 2.714	50
***A. Sativum***					
Cd	0.444 ± 0.007	0.459 ± 0.004	0.495 ± 0.012	0.457 ± 0.011	0.5
Co	0.402 ± 0.028	0.442 ± 0.017	0.411 ± 0.033	0.463 ± 0.022	1
Cr	0.298 ± 0.158	0.169 ± 0.004	0.381 ± 0.159	0.272 ± 0.123	50
Cu	1.152 ± 0.008	1.151 ± 0.005	1.130 ± 0.020	1.189 ± 0.002	20
Fe	0.192 ± 0.003	0.311 ± 0.023	0.966 ± 0.657	0.187 ± 0.012	10
Mn	2.949 ± 0.534	3.472 ± 0.900	3.458 ± 0.940	5.682 ± 0.150	1000
Pb	0.327 ± 0.050	0.052 ± 0.0192	0.501 ± 0.162	0.488 ± 0.188	30
Zn	16.04 ± 0.031	18.28 ± 0.804	9.348 ± 3.22	4.535 ± 0.571	50

Source: From [33], MPL: maximum permissible limit, SE: Standard Error, C control, PM poultry waste, P press mud, FYM farmyard manure.

**Table 2:** Analysis of variance (*p* values) of heavy metal concentrations in soil and *A. sativum.*

Metals	Soil	Plant
Cd	5.516 *	0.003 **
Co	1.163 ns	0.005 *
Cr	2.114 ***	0.046 *
Cu	2.372 *	0.004 **
Pb	12.984 **	0.831 *
Fe	60.111ns	8.918 **
Mn	2.724 *	0.262 **
Zn	711.514 **	237.171 ***

** significant at 0.001 level, * significant and *** highly significant, ns = non-significant.

**Table 3:** Bioconcentration factor for *A. sativum*/soil.

Metals	Treatments			
	Control	PW	PM	FYM
Cd	1.9103	5.40	3.245902	1.322338
Co	0.069	0.984	0.309573	0.18898
Cr	0.437	2.608	1.970213	0.788406
Cu	1.68	1.23	0.192482	0.305656
Pb	3.58	3.273	25.76	3.351254
Fe	0.172	1.628136	1.199653	1.592935
Mn	1.900	0.034899	1.027692	2.076
Zn	2.40	0.80952	2.002892	0.428801

**Table 4:** Pollution load index for heavy metals in soil grown with *A. sativum.*

Metals	Treatments			
	C	PW	PM	FYM
Cd	0.1560	0.057	0.102	0.231
Co	0.6387	0.049	0.238	0.269
Cr	0.455	0.043	0.156	0.23
Cu	0.0813	0.110	0.700	0.463
Pb	0.0006	0.037	0.050	0.062
Fe	0.2998	0.011	0.010	0.005
Mn	0.1509	0.511	0.105	0.239
Zn	0.3861	0.226	0.099	0.511

**Table 5:** Daily intake and health risk index for consumption of *A. sativum.*

Metals	Treatments	C	PW	PM	FYM
**Cd**	DIM	0.0025	0.0026	0.0028	0.00267
	HRI	2.5972	2.6849	2.8944	2.67228
**Co**	DIM	0.0023	0.0025	0.0028	0.00270
	HRI	0.0547	0.0602	0.0912	0.06296
**Cr**	DIM	0.0017	0.0009	0.0027	0.00159
	HRI	0.0011	0.0006	0.0018	0.00106
**Cu**	DIM	0.0067	0.0067	0.0066	0.00693
	HRI	0.1685	0.1683	0.1653	0.17381
**Pb**	DIM	0.0011	0.0018	0.0056	0.00109
	HRI	0.3222	0.5195	1.6138	0.31242
**Fe**	DIM	0.0172	0.0203	0.0202	0.03322
	HRI	0.0246	0.0290	0.0288	0.04746
**Mn**	DIM	0.0019	0.0003	0.0546	0.00285
	HRI	0.0246	0.0074	0.0714	0.06959
**Zn**	DIM	0.0938	0.1069	0.0546	0.02651
	HRI	0.2535	0.2889	0.1477	0.07167

## Data Availability

Data and material are available for research purposes and for reference.
